# State-of-the-Art Imaging Techniques in Metastatic Spinal Cord Compression

**DOI:** 10.3390/cancers14133289

**Published:** 2022-07-05

**Authors:** Tricia Kuah, Balamurugan A. Vellayappan, Andrew Makmur, Shalini Nair, Junda Song, Jiong Hao Tan, Naresh Kumar, Swee Tian Quek, James Thomas Patrick Decourcy Hallinan

**Affiliations:** 1 Department of Diagnostic Imaging, National University Hospital, 5 Lower Kent Ridge Rd, Singapore 119074, Singapore; andrew_makmur@nuhs.edu.sg (A.M.); shalini_nair@nuhs.edu.sg (S.N.); junda_song@nuhs.edu.sg (J.S.); swee_tian_quek@nuhs.edu.sg (S.T.Q.); james_hallinan@nuhs.edu.sg (J.T.P.D.H.); 2 Department of Radiation Oncology, National University Cancer Institute Singapore, National University Hospital, Singapore 119074, Singapore; bala_vellayappan@nuhs.edu.sg; 3 Department of Diagnostic Radiology, Yong Loo Lin School of Medicine, National University of Singapore, 10 Medical Drive, Singapore 117597, Singapore; 4 University Spine Centre, Department of Orthopaedic Surgery, National University Health System, 1E Lower Kent Ridge Road, Singapore 119228, Singapore; jonathan_jh_tan@nuhs.edu.sg (J.H.T.); dosksn@nus.edu.sg (N.K.)

**Keywords:** metastatic spinal cord compression, metastatic epidural spinal cord compression, CT, MRI, metal artifact reduction, stereotactic body radiotherapy, stereotactic radiosurgery, image-guided radiotherapy, deep learning, Bilsky scale

## Abstract

**Simple Summary:**

Metastatic Spinal Cord Compression (MSCC) is a feared complication in oncology patients due to its potential for severe pain, permanent neurological disability and mechanical instability of the spine. This narrative review, conducted by keyword searches in PubMed and Google Scholar databases, aims to describe the important role of imaging in MSCC diagnosis and treatment. Diagnosis is typically achieved via Magnetic Resonance Imaging (MRI), although Computed Tomography (CT) Myelogram and conventional CT imaging can be performed in certain clinical situations. Metal artifact reduction techniques for MRI and CT are continually being researched to facilitate imaging in MSCC patients with spinal implants. Imaging also has an important role in pre-treatment planning, in-room image-guidance, and post-treatment follow-up for MSCC patients treated with stereotactic body radiotherapy. Recent advances in deep learning tools for image analysis can reduce the time to MSCC diagnosis, enabling earlier treatment for superior functional outcomes.

**Abstract:**

Metastatic Spinal Cord Compression (MSCC) is a debilitating complication in oncology patients. This narrative review discusses the strengths and limitations of various imaging modalities in diagnosing MSCC, the role of imaging in stereotactic body radiotherapy (SBRT) for MSCC treatment, and recent advances in deep learning (DL) tools for MSCC diagnosis. PubMed and Google Scholar databases were searched using targeted keywords. Studies were reviewed in consensus among the co-authors for their suitability before inclusion. MRI is the gold standard of imaging to diagnose MSCC with reported sensitivity and specificity of 93% and 97% respectively. CT Myelogram appears to have comparable sensitivity and specificity to contrast-enhanced MRI. Conventional CT has a lower diagnostic accuracy than MRI in MSCC diagnosis, but is helpful in emergent situations with limited access to MRI. Metal artifact reduction techniques for MRI and CT are continually being researched for patients with spinal implants. Imaging is crucial for SBRT treatment planning and three-dimensional positional verification of the treatment isocentre prior to SBRT delivery. Structural and functional MRI may be helpful in post-treatment surveillance. DL tools may improve detection of vertebral metastasis and reduce time to MSCC diagnosis. This enables earlier institution of definitive therapy for better outcomes.

## 1. Introduction

Metastatic spinal cord compression (MSCC) refers to compression of the spinal cord and/or cauda equina by metastatic disease or direct extension of metastatic disease from the vertebra. Typically, it is due to compression of the thecal sac by an epidural tumour, although it may rarely be caused by intradural or intramedullary metastasis. It is a feared complication in oncology patients due to its potential for severe pain, mechanical instability of the spine and permanent neurological deficits including weakness, sensory loss, and bladder and bowel dysfunction. Prompt recognition and early treatment are necessary for pain relief and preservation of neurological function [1,2,3,4,5].

The spine is the most common site of skeletal metastasis affecting up to 40% of cancer patients, of which up to 20% may become symptomatic from spinal cord compression [4,6]. The annual prevalence of symptomatic MSCC has been estimated to be approximately 3–5% [2,4,7] and the thoracic spine is most frequently involved [3,4]. It is the initial presentation of malignancy in approximately 20% of patients [8,9]. Metastasis from any primary cancer may involve the spine, and the most frequently encountered are lung, prostate and breast cancers [3,4,8,9,10].

In a multicentre study involving lung cancer patients between May 2006 and May 2007, treatment costs for symptomatic bone metastasis and skeletal-related events including MSCC ranged from 374 €–4672 € a month [11]. This equates to up to 56,064 € a year per patient, evidently a major burden on healthcare expenditure. With improving cancer treatment including the development of novel targeted therapies, cancer patient survival and consequently incidence of MSCC are expected to rise [6].

MSCC is a time-sensitive diagnosis and is considered an oncological emergency [3,5]. If there are delays to treatment and compression is not relieved quickly, progressive irreversible neurological dysfunction can occur. This further affects prognosis as the patient’s pre-treatment neurological status and duration of ambulatory loss are major determinants for regaining ambulation post treatment [1,5,12,13].

Diagnosis of MSCC is suggested by the presence of back pain and/or neurological deficits including weakness, sensory loss, ataxia and cauda equina syndrome. Back pain that is exacerbated by straining efforts (for example, coughing and sneezing) or nocturnal back pain should be viewed with suspicion [1,2], and the presence of movement-related pain suggests mechanical instability [2,14]. Unfortunately, a convincing history of back pain may be difficult to elucidate due to overlapping symptoms with pre-existing conditions like spondylosis or masked due to the use of analgesics/opiates. Moreover, these symptoms are nonspecific and delays to diagnosis are frequently encountered [1]; evidence from a Scottish audit conducted from 1997–1999 showed that the median times to referral from the onset of back and radicular pain were estimated to be 3 months and 9 weeks respectively [15]. Another study by van Tol et al. (2021) noted a median delay of 99 days from the onset of symptoms to definitive treatment [16]. This is considerably longer than the recommended timeframe of 48 h from initial clinical suspicion of MSCC to definitive treatment, as stipulated by the 2008 National Institute for Health and Care Excellence (NICE) guidelines [1].

Magnetic Resonance Imaging (MRI) is the most accurate diagnostic imaging modality and should be performed within 24 h of clinical suspicion of MSCC [1,2]. The management of MSCC is highly individualized, with a treatment strategy devised after consultation with various specialties including the oncologist, spine surgeon, radiologist and radiation oncologist [1,12]. The Neurological, Oncological, Mechanical and Systemic (NOMS) framework was developed in 2013 by the Memorial Sloan-Kettering Cancer Centre to guide management decisions in response to the numerous new treatment options for MSCC. It is a decision framework that considers Neurological, Oncological, Mechanical and Systemic factors to arrive at an optimal treatment strategy for patients with MSCC [17]. Based on this framework, patients with radioresistant tumours causing high-grade MSCC and patients who have mechanical instability of the spine will be treated surgically if they do not have comorbidities or other factors that preclude operative management [6,17]. Aside from surgery, other modes of local treatments include radiotherapy such as conventional external beam radiotherapy (cEBRT) or stereotactic body radiotherapy (SBRT). Exquisitely chemo-sensitive conditions, such as lymphoma and small cell carcinoma, may be managed with systemic therapy upfront [12].

Deciding the optimal treatment strategy may not always be straightforward. An example would be the initial treatment of spinal Ewing sarcoma causing neurological compromise. The rarity of this condition limits the feasibility of conducting high-powered clinical trials. While upfront surgery relieves decompression, the risk of tumour dissemination is uncertain. Chemotherapy, on the other hand, reduces micrometastasis and is the initial treatment of choice for Ewing sarcoma occurring in other regions of the body. A previous literature review has noted no statistically significant difference between these two treatment choices in terms of disease recurrence and overall survival [18].

The 2008 NICE guidelines have set out recommendations for timely MSCC diagnosis and management. A more updated version is in progress, and it is slated for publication on 23 August 2023 [19]. A dedicated referral pathway has also been proposed by a team in the United Kingdom to expedite access to definitive treatment for patients with MSCC. This pathway, led by an MSCC coordinator and with representatives from the clinical oncology and neurosurgical team, will improve communication among the specialties involved, expedite referrals and review of relevant radiological imaging, amongst other time-saving measures. Including this pathway in the medical school curriculum has been suggested for better awareness and knowledge among junior doctors on the time-sensitive nature of MSCC diagnosis and treatment [20].

## 2. Aims of This Study

The remainder of this narrative review will discuss the following:

1. The indications, strengths and limitations of various imaging modalities for diagnosis of MSCC, focusing primarily on MRI and including techniques for metal artifact suppression on MRI and CT;
2. The role of imaging in SBRT with regards to treatment planning, image-guidance during treatment and post-treatment follow-up;
3. Recent advances in deep learning (DL) tools for image acquisition and analysis, which hold potential to reduce time to MSCC diagnosis, enabling more efficient patient referrals and treatment selection.

A flow diagram of the search methods of this narrative review is included as supplementary material (Figure S1).

## 3. Imaging Modalities for Diagnosis of Metastatic Spinal Cord Compression (MSCC)

### 3.1. Goals of Imaging

**Figure 1:**
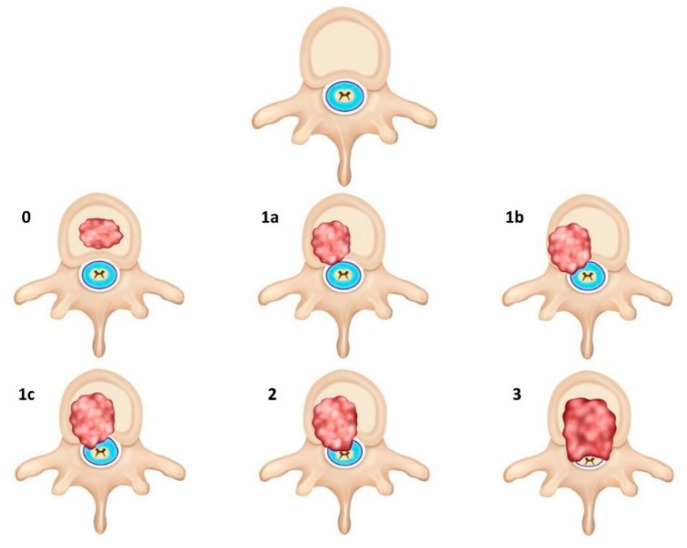
Metastatic spinal cord compression is classified with a six-point scale, also referred to as the Bilsky grading scale. Legend: red = tumour; purple line = dura; light blue = cerebrospinal fluid (CSF); yellow = spinal cord. The gradings are as follows—Bilsky 0: tumour that is confined to the bone (i.e., without epidural involvement); Bilsky 1a: tumour with epidural involvement but without indentation of the thecal sac; Bilsky 1b: tumour with epidural involvement and indentation of the thecal sac but without spinal cord contact; Bilsky 1c: tumour with epidural involvement and spinal cord contact without cord compression; Bilsky 2: tumour with epidural involvement and compression of the spinal cord but without obliteration of the surrounding CSF spaces; Bilsky 3: tumour with epidural involvement and severe compression of the spinal cord with complete obliteration of the surrounding CSF spaces [2,21].

The goals of imaging in MSCC are as follows:

1. Confirm the diagnosis of MSCC;
2. Identify the level of involvement (especially because localization by clinical examination may not be reliable) [2] as well as areas of epidural and paraspinal involvement;
3. Assess for other sites of metastatic disease in the vertebral column, which has implications for both prognosis and treatment planning [2];
4. Radiologically grade the degree of MSCC. This is achieved via a six-point grading scale developed by the Spine Oncology Study Group (SOSG), also known as the Bilsky Grading Scale [21] (Figure 1);
5. Determine the presence of mechanical instability. This is achieved via the Spine Instability Neoplastic Score (SINS) which was also developed by the SOSG [22] (Table 1). It uses radiologic criteria and pain characteristics to identify patients at high risk of spinal instability from underlying neoplasm.

### 3.2. Magnetic Resonance Imaging (MRI)

With its superior soft tissue characterization, MRI remains the gold standard of imaging for MSCC diagnosis [1,2]. Recommended sequences include sagittal pre-and post-contrast T1-weighted, T2-weighted and sagittal short tau inversion recovery (STIR) sequences. Where significant abnormalities are detected, dedicated axial sequences are performed [1,2]. In our institution, this includes both axial T2-weighted and post-contrast T1-weighted sequences. Table 2 shows the typical parameters used in our institution for an MRI of the whole spine for MSCC assessment.

**Figure 2:**
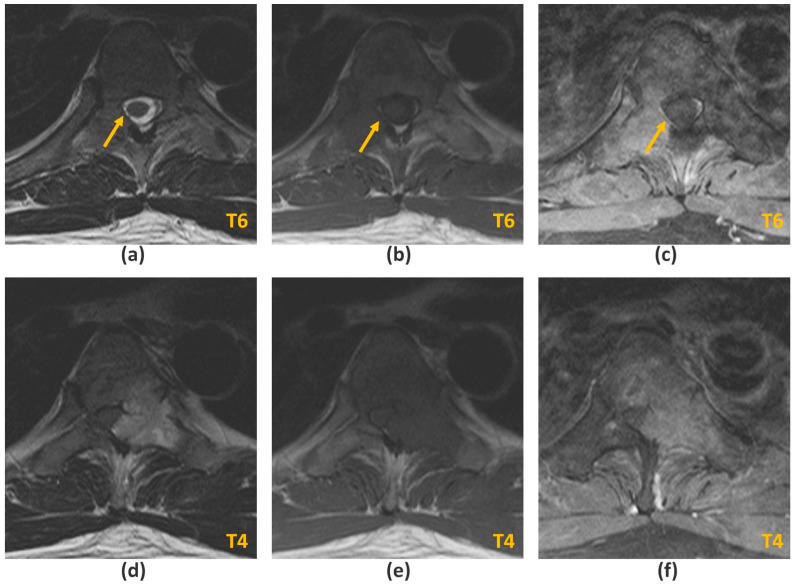
Axial T2-weighted (**a**), pre-contrast T1-weighted (**b**) and post-contrast fat-suppressed T1-weighted (**c**) MR images of a lung carcinoma patient with metastatic spinal cord compression (MSCC) at the level of T6. Epidural tumour extension is noted with thecal sac indentation but no spinal cord abutment (solid arrows), best demonstrated on the T2-weighted sequence (Bilsky 1b). Marrow infiltration by the tumour is indicated by hypointensity on the T1-weighted sequence, with corresponding enhancement seen on the post-contrast sequence. Axial T2-weighted (**d**), pre-contrast T1-weighted (**e**) and post-contrast fat-suppressed T1-weighted (**f**) MRI images of the same patient at a higher level (T4) show high-grade MSCC with complete obliteration of the CSF spaces, again best demonstrated on the T2-weighted sequence (Bilsky 3). Prominent enhancement of the epidural and vertebral component of the tumour is seen in post-contrast images.

The ability of MRI to evaluate for spinal cord and nerve root compression is best demonstrated on T2-weighted sequences due to the excellent contrast between the high signal cerebrospinal fluid (CSF), low signal spinal cord and iso-hyperintense tumour (usually a lower signal intensity than CSF) (Figure 2a,d). This produces a ‘myelogram-like effect’, allowing optimal visualization of the presence or obliteration of CSF spaces separating the tumour and spinal cord [21]. In addition, cord signal changes including myelomalacia and oedema are best assessed on T2-weighted sequences.

T1-weighted (Figure 2b,e) and STIR are the two most important sequences for identifying metastatic disease. The former is the only imaging modality capable of direct visualization of bone marrow [23]. This enables MRI to detect early fatty marrow replacement by metastatic spread even before cortical destruction, which is an advantage over CT [23,24]. Iso- or hypointensity of marrow lesions relative to adjacent skeletal musculature or non-degenerated intervertebral discs have reported sensitivities and specificities of 94–100% and 92–94% respectively in detecting marrow infiltrative lesions [25]. On STIR imaging, metastatic deposits are generally hyperintense, save for highly sclerotic lesions which may demonstrate reduced or absent oedema. In such cases, T1-weighted sequences are more reliable [26].

The presence of intravenous contrast is useful for detecting enhancing vertebral metastasis and is particularly important for identifying the epidural component that is compressing the thecal sac in MSCC (Figure 2c,f). It can also delineate the extent of foraminal and paraspinal tumour extension as well as identify sites of leptomeningeal/intramedullary disease [2]. Post-contrast fat suppression is important as marrow fat is intrinsically T1 hyperintense. Table 3 summarizes the advantages and disadvantages of the various MRI sequences used in MSCC diagnosis.

The diagnosis of MSCC relies on the demonstration of thecal sac compression by tumour on MRI. This is quantified by a six-point grading scale (Bilsky grading scale) developed by the SOSG, which had previously shown high inter- and intra-rater reliability among seven spine surgeons [21] (Figure 1). It relies on axial T2-weighed sequences. Low-grade disease is indicated by Bilsky 0, 1a and 1b while high-grade disease is indicated by Bilsky 2 and 3. This dichotomization is important as radiotherapy (including SBRT and stereotactic radiosurgery) can be considered for initial treatment for low-grade disease while surgical decompression should be considered prior to radiotherapy for high-grade disease. The management of Bilsky 1c MSCC remains to be clearly defined [6,12,17].

MRI is also useful for assessing the presence of spinal instability, which affects the definitive treatment of MSCC. The SINS was developed by the SOSG (Table 1) and relies on pain characteristics as well as imaging findings on MRI and CT like the presence of subluxation/translation, extent of vertebral body compression and nature of the metastatic deposit (osteolytic, osteoblastic or mixed). A score of seven or higher suggests significant spinal instability and a surgical consult is recommended [2,22]. Multiple validation studies involving various specialty doctors including radiologists and radiation oncologists have reported moderate to high intra- and interobserver agreement [27,28,29].

MRI has been shown to have high specificity and sensitivity in diagnosing MSCC, with a prospective evaluation of 70 patients previously demonstrating sensitivities of 73–92% and specificities of up to 90%, exceeding or comparable to diagnosis by myelography [30]. More recent studies have cited comparable sensitivities and specificities of 93% and 97% respectively in diagnosing MSCC [20,31].

In addition to imaging the site of clinical suspicion, attention should be made to imaging the remainder of the spine as additional sites of metastatic disease/MSCC have been reported to occur in a quarter to a third of cases [3,32]. A retrospective study of 337 MSCC cases at Mayo Clinic from 1985 to 1993 showed that failure to include the thoracic or lumbosacral spine in the field of imaging when the symptomatic lesion was sited elsewhere, had resulted in missed lesions in 21% of cases [9].

#### 3.2.1. Challenges of MRI: Metal-Related Artifacts

MSCC patients with spinal instability or high-grade disease may undergo surgical intervention with spinal instrumentation. Imaging around metal is a perennial problem when performing follow-up studies for these patients. The presence of metal-related artifacts obscures relevant anatomy and disease on both MR and CT imaging. It also poses issues with radiation treatment planning and precise dose delivery to the target area [33,34].

Metal-related artifacts arise due to the paramagnetic and ferromagnetic properties of metal implants. These result in marked variability in the local magnetic field near the implant which causes alterations in proton spin frequencies in these areas compared to metal-free tissues. These have several effects on the final image. Firstly, due to accelerated intravoxel dephasing, signal losses may occur. Secondly, the altered frequencies result in spatial misregistration. When this occurs in the frequency-encoding direction, the effects on the final image include signal loss (abnormal areas of low signal intensity), signal pile-up (abnormal areas of high signal intensity) and in-plane geometric distortion due to spatial misinterpretation. When this occurs in the slice-encoding direction, through-plane geometric distortion occurs. Thirdly, there is reduced efficacy of spectral-based fat suppression, as these are contingent on the homogeneity of the local magnetic field to exploit the subtle chemical shift differences of water and fat. All these effects contribute to extensive metal-related artifacts around the implant that may preclude meaningful interpretation of images in that region [33,34,35,36,37].

Numerous factors affect the extent of the metal-related artifact. These can broadly be classified into implant-related factors and hardware factors. Implant-related factors include the implant size, configuration, positioning and material composition. A rounded and symmetrical implant shape is favoured as sharp edges or complex shapes cause more extensive artifacts [35]. Artifacts are also reduced when the long axis of the implant is parallel to the main magnetic field [35].

**Figure 3:**
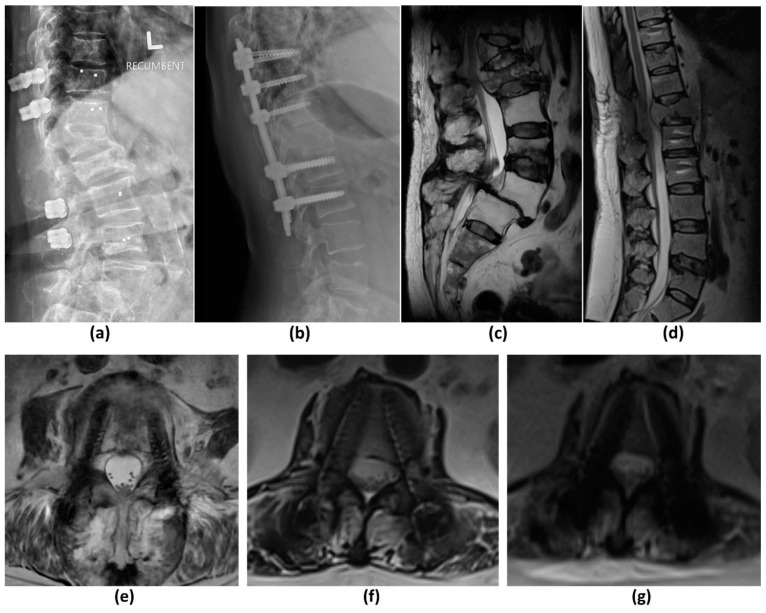
Lateral radiograph (**a**), sagittal (**c**) and axial (**e**) T2-weighted metal artifact reduction sequence (MARS) MR image of a patient with CFR-PEEK screw implants in the spine. Lateral radiograph (**b**), sagittal (**d**) and axial (**f**) T2-weighted MARS (“WARP”) MR image of a patient with titanium screw implants in the spine. The titanium screws are partially visualized in (**d**) due to the slice orientation and are best seen in the T9, L1 and L2 vertebral bodies. In contradistinction to the titanium implants, CFR-PEEK implants are radiolucent on radiographs and result in less metal-related artifacts on MRI. Axial T2-weighted image of the patient with titanium implants without MARS demonstrate significantly increased geometric distortion and signal losses, rendering assessment of the vertebral body and contents of the spinal canal difficult (**g**).

Implant material composition has a significant bearing on artifacts and distortions in both in-plane and through plane-directions. Stainless steel produces the greatest susceptibility, followed by cobalt-chromium and titanium [35,36,37,38]. The use of carbon fibre reinforced polymer (C-FRP) implants, for example, carbon fibres with reinforced polyetheretherketone (CFR-PEEK) have shown promise in this field (Figure 3a–f). In addition to being radiolucent and nonmagnetizable, these have favourable characteristics suitable for use as an orthopaedic implant including low weight, good mechanical strength and improved load transfer to bone, thereby reducing stress-shielding [37,39,40,41]. In a 2015 qualitative assessment of metal artifacts in patients with femoral or tibial intramedullary nails, CFR-PEEK implants showed reduced metal-related artifacts on T1-weighted, STIR and contrast-enhanced fat-suppressed T1-weighted imaging compared to titanium implants, with high intraobserver agreement [42]. A prior study has also demonstrated CFR-PEEK implants to have more significant suppression of metal artifacts than certain metal artifact reduction (MAR) techniques on MR [43].

Hardware factors refer to the field strength of the MRI scanner, sequence parameters as well as the use of certain metal-artifact reduction sequences or reconstruction techniques.

Metal artifact reduction sequences (MARS) is a general term and may describe measures taken to optimize imaging around metal on MRI, rather than referring to a specific pulse sequence [35,36]. Conventional MARS techniques include using a 1.5T instead of a 3T scanner, increasing the receiver bandwidth and matrix size, thin sections, decreasing echo times, and utilizing fast spin-echo (FSE) sequences (rather than gradient-echo sequences). As misregistration artifacts are more pronounced in the frequency-encoding direction than phase-encoding direction, adjusting these may help to reduce artifacts in the region of interest [33,35,36,37]. Increasing echo train lengths have previously been thought to be effective at reducing metal-related artifacts, however a recent study in 2017 suggests that this may in fact result in increased image degradation around the implant [44]. It is important to note that many of these conventional measures result in a reduced signal-to-noise ratio (SNR), which may necessitate longer scan times.

For fat suppression, STIR sequences are the technique of choice as they are more resistant to magnetic field heterogeneity. Drawbacks include reduced SNR and an inability to image post-contrast administration as the contrast-enhanced tissues also demonstrate a T1 shortening effect resulting in nullification of their signal. Dixon turbo spin echo (TSE) sequences can also be considered due to their relative resistance to heterogeneities in the local magnetic field. Unlike STIR sequences, these are applicable in post-contrast sequences and do not demonstrate a reduced SNR. However, they are less effective at homogeneous fat suppression around metal [33,35,36,37]. Table 4 summarizes the advantages and disadvantages of several fat-saturation sequences that have been described in this paper.

Specific MARS techniques have been developed in addition to the conventional techniques just described. These include “WARP”, Slice-Encoding for Metal Artifact Correction (SEMAC) and Multi-Acquisition Variable Resonance Image Combination (MAVRIC) sequences [35,36,37].

“WARP” utilizes conventional MARS with multi-directional View-Angle Tilting (VAT) (Figure 3f,g). VAT is a technique used to reduce in-plane artifacts by employment of an additional gradient in the slice-selection direction concurrent with the conventional readout gradient. This has a shearing effect on the pixels of interest and tilts the readout gradient at an angle, which reduces in-plane artifacts including signal loss and pile-up. However, it comes at a drawback of increased blurring. SEMAC is a two-dimensional TSE based sequence which uses phase encoding in the third dimension (slice selection) to identify distortions for each slice. This information from all the overlapping sections is used during post-processing to identify how through-plane distortion has affected the image and corrects for it. However, scanning time is invariably increased. MAVRIC is an FSE-based three-dimensional acquisition technique which utilizes a series of frequency-selective excitations together with a multispectral VAT-type readout, to reduce through-plane and in-plane artifacts. Scanning time is increased, as well as the specific absorption rate (SAR). Through-plane aliasing artifacts are also encountered particularly in the hip and shoulder joints [35,36,37,45,46,47].

MAVRIC-SL is a relatively recent hybrid acquisition sequence which combines the techniques from MAVRIC and SEMAC to reduce both in-plane and through-plane distortions [35,46,47]. Previously, MAVRIC and SEMAC sequences were performed on 1.5T machines due to the increased susceptibility artifacts on 3T machines [47]. However. several studies evaluating the performance of MAVRIC-SL on 3T machines have been promising, demonstrating significantly reduced metal-related artifacts, improved image quality and improved visualization of the bone-implant interface compared to conventional FSE/FSE-STIR sequences. In one study involving 19 patients, the inclusion of MAVRIC-SL in the imaging protocol had ascertained the need for and type of surgery in five patients, as well as negated the need for surgical intervention in 13 patients [45,46,47,48].

While promising for MSCC patients with spinal instrumentation, the aforementioned MARS techniques are beset by prolonged scan times, similar to conventional MARS techniques. Longer scanning times can increase motion artifacts and may limit the feasibility of obtaining sequences in all three orthogonal planes [47]. It is also problematic for MSCC patients who frequently have recumbent pain. Techniques to reduce scan times are subsequently discussed.

**Table 4:** Advantages and disadvantages of several fat-saturation sequences [35,37,49,50,51]. CHESS, chemical shift selective fat saturation. SNR, signal-to-noise ratio. STIR, short tau inversion recovery. SAR, specific absorption rate. TSE, turbo spin echo. FOV, field-of-view.

Title 1	Advantages	Disadvantages
Spectral fat-saturation/ CHESS	Versatile, can be applied to any pulse sequence including post-contrast sequences; High SNR; Relatively fast technique; Relatively high resolution	Sensitive to magnetic field inhomogeneities^1^, especially around metal implants
STIR	Less sensitive to magnetic field inhomogeneities ^1^	Reduced SNR; Relatively long imaging times; Relatively low resolution; High SAR; Inability to image post-contrast administration (other materials with a short T1 relaxation time including protein, melanin, and methaemoglobin would also be suppressed)
Dixon TSE	Less sensitive to magnetic field inhomogeneities ^1^; Ability to image post-contrast administration; SNR improved compared to STIR sequences	Less effective than STIR at homogeneous fat suppression around metal implants; Relatively long imaging times; Fat-water swap may occur

^1^ These include magnetic field inhomogeneities introduced by metal implants, challenging geometry of the imaged anatomy, off-centre imaging, and a large FOV.

#### 3.2.2. Challenges of MRI: Long Scan Acquisition Times

In select MSCC patients, a bolus of glucocorticoids or general anaesthesia may be considered for pain relief to allow the patient to lie still during prolonged scan times [2]. Glucocorticoids reduce inflammation and vasogenic edema of the spinal cord, in addition to their cytolytic properties in steroid-responsive malignancies. These result in pain relief, protection against cord ischemia/infarction and a possible temporary improvement of neurological function while awaiting definitive treatment [2,12,52].

In other patients, a recommended practical scan time of up to five minutes is suggested for patient comfort, reduction of motion artifact and efficient scanner utilization [53,54]. There is considerable ongoing research on the feasibility of coupling specific MARS techniques with standard-fast imaging approaches. For example, SEMAC has been shown to be successfully combined with acceleration techniques like parallel imaging, standard echo-train imaging, and partial-Fourier imaging, with no reduction in its ability to reduce metal-related artifacts [54]. However, it should be noted that parallel imaging may be less feasible in spine patients because of incompatible coil array sensitivity variation. Other advanced acceleration techniques include compressed sensing (CS), which exploits the inherent sparsity of MRI acquisitions to decrease the amount of phase encoding steps and therefore imaging times [53,55,56]. As there are two phase-encoding dimensions in k-space in SEMAC acquisitions, inherent sparsity is significant and CS can be applied. CS-SEMAC reportedly has an 8-fold acceleration of k-space encoding without reduction in image quality as compared to original SEMAC images [35,53]. This extent of time savings had not been achieved with combinations of parallel and partial-Fourier imaging and has allowed the development of other protocols like STIR to maximize the diagnostic quality of the study [53]. Another study on 13 patients with spinal implants had shown that CS-multispectral imaging had equivalent or better image quality than original multispectral imaging, with equivalent or better nerve visualization [55].

Recent advances in DL assisted image acquisition and reconstruction like AIR^TM^ Recon DL (GE Healthcare, Waukesha, WI, USA) may also be able to shorten scan times via an improvement in SNR [57].

While more research on the combination of acceleration techniques with specific MARS is still needed, these initial studies are promising for MSCC patients with spinal instrumentation who require regular imaging follow-up. A reduction in MR scan times is also beneficial for SBRT planning and treatment.

#### 3.2.3. Challenges of MRI: Others

**Figure 4:**
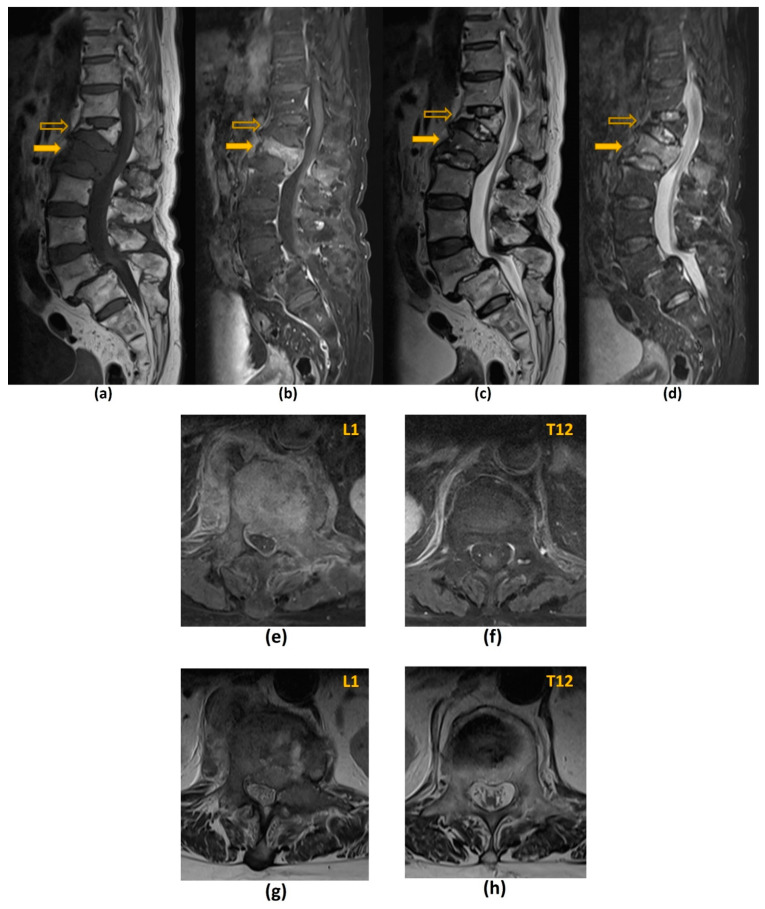
Sagittal pre-contrast T1-weighted (**a**), post-contrast fat-suppressed T1-weighted (**b**), T2-weighted (**c**) and STIR (**d**) sequences of a 70-year-old male patient with lung cancer that had metastasized to the spine. Axial post-contrast fat-suppressed T1-weighted sequences at the level of L1 (**e**) and T12 (**f**), and axial T2-weighted sequences at the level of L1 (**g**) and T12 (**h**) of the same patient. There is a pathological L1 compression fracture (solid arrow) with diffuse fatty marrow replacement extending to the posterior elements by the tumour. A convex posterior border is demonstrated as well as an enhancing epidural component. This causes low-grade (Bilsky 1c) MSCC. At the level of T12 (open arrow), no significant marrow replacement is seen and the posterior elements return normal signal intensities. There is subtle retropulsion of fracture fragments and no enhancing epidural component. These findings suggest a T12 osteoporotic compression fracture.

Pathological vertebral body fracture/collapse is known to result in MSCC [5,58] and it is important to distinguish benign osteoporotic compression fractures from malignant vertebral compression fractures (VCF) on imaging due to the significant implications on treatment (Figure 4). MR findings that have been shown to suggest a malignant aetiology include an expansile convex posterior cortex, marrow signal abnormalities of the posterior elements, destruction of the pedicles, heterogeneously increased enhancement of the vertebral body, abnormal epidural or paraspinal soft tissue or enhancement and the presence of other spinal metastases. MR findings shown to favour a benign osteoporotic aetiology include the presence of a T1-weighted and T2-weighted hypointense band thought to represent cancellous bone compaction, fluid or gas-filled clefts, posterior retropulsion of bony fragments, normal marrow signal intensity (or a well-demarcated regular margin separating the spared marrow and abnormal marrow within the fractured vertebra) and the presence of multiple compression fractures (with the notable exception of multiple myeloma). Also, VCF occurring in the thoracic and lumbar spine are reportedly more likely to be malignant than those in the cervical spine, though the clinical utility of this finding remains to be established [24,26,59,60]. Table 5 summarises the characteristics that may distinguish a benign osteoporotic compression fracture from a malignant VCF.

A recent retrospective study [61] however, disputes the ability of MRI to accurately distinguish between malignant and benign VCF, reporting only moderate interobserver agreement and moderate concordance with the reference standard (biopsy findings or follow-up of more than 6 months). The MR images in the study were reviewed by 25 clinicians including neurosurgeons, radiologists, orthopaedic surgeons and radiation oncologists. Newer studies have shown the possible role of DWI, perfusion/dynamic contrast-enhanced (DCE) imaging and opposed-phase MRI in distinguishing between these two entities [24,26,60]. Future research is needed for further validation of these findings.

Finally, a limitation in using signal abnormalities to distinguish between benign and malignant VCF is that marrow signal abnormalities may be influenced by other factors like radiation-induced fatty marrow replacement. Granulocyte colony-stimulating factor (GCSF) in chemotherapeutic regimens has also been shown to induce diffuse marrow signal changes including T1 hypointensity and STIR hyperintensity, mimicking metastatic deposits [62,63].

### 3.3. Computed Tomography (CT) Myelogram

**Figure 5:**
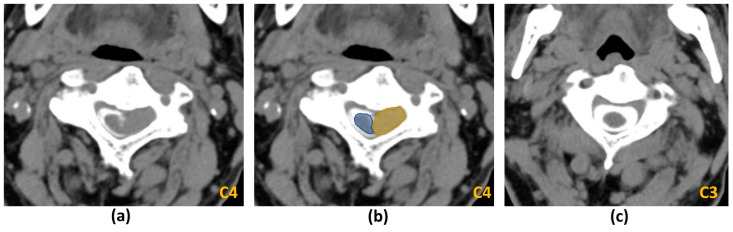
CT Myelogram of a lung cancer patient with suspected metastatic spinal cord compression (MSCC) at the level of C4, without annotations (**a**) and with annotations (**b**). The epidural disease is indicated by the yellow colourwash and the spinal cord by the blue colourwash in (**b**). At this level, the epidural tumour abuts the spinal cord with partial obliteration of the contrast-opacified subarachnoid space (Bilsky 2 MSCC). More superiorly at the level of C3 (**c**), no evidence of MSCC or epidural disease is seen.

Computed Tomography (CT) Myelogram involves intrathecal administration of iodinated contrast followed by volumetric thin section (<3 mm) CT imaging without intravenous contrast administration. Multiplanar sagittal and coronal reformations are performed in both soft tissue and bone algorithms [2]. Intrathecal contrast allows for excellent resolution between the CSF, tumour and spinal cord (Figure 5).

The role of CT Myelogram in MSCC diagnosis has decreased since the advent of MRI given the latter’s superior soft tissue resolution, ability to assess for cord signal changes, non-invasiveness and reduced exposure to ionizing radiation [64]. Nevertheless, CT myelogram remains an important tool in the evaluation of spinal pathology including MSCC in the following clinical situations [1,2,23,64]:

1. MRI is contraindicated (e.g., due to extreme claustrophobia, large body habitus, inability to lie still for a prolonged period of time, metallic foreign body in orbit, or a noncompatible cardiac device);
2. Poor diagnostic yield of MRI due to metal artifacts from spinal implants (CT will also require MAR techniques);

CT myelogram also has added benefits as CSF can be obtained for cytology and other analyses.

It is advised to perform CT myelogram only in institutions where neurosurgical expertise is available due to the potential for neurological deterioration post lumbar puncture below a complete spinal block. This is thought to be related to downward spinal coning and reportedly affects at least 14% of patients. A spinal block also limits the evaluation of superior aspects of the spine, and occasionally an additional cervical approach of intrathecal contrast administration is necessary [2,65].

The sensitivity and specificity of modern CT myelogram compared to MRI have not been formally evaluated [2], with previous studies mainly focusing on the diagnostic performance of MRI compared to myelography [30,66,67,68]. However, CT myelogram appears to have comparable sensitivity and specificity to contrast-enhanced MRI for MSCC diagnosis [2].

### 3.4. Conventional CT Imaging

CT has the ability to characterize vertebral metastasis, and it plays a complementary role to MRI in this regard. Due to its superior resolution and detailed assessment of bone cortical anatomy, CT can detect cortical destruction, assess the nature of metastatic lesions (osteolytic or osteoblastic) including matrix mineralization, and provide better visualization of sclerotic lesions and pathological fractures compared to MRI. CT imaging can reportedly detect metastatic lesions up to 6 months earlier than radiographs [23,24,26,69].

**Figure 6:**
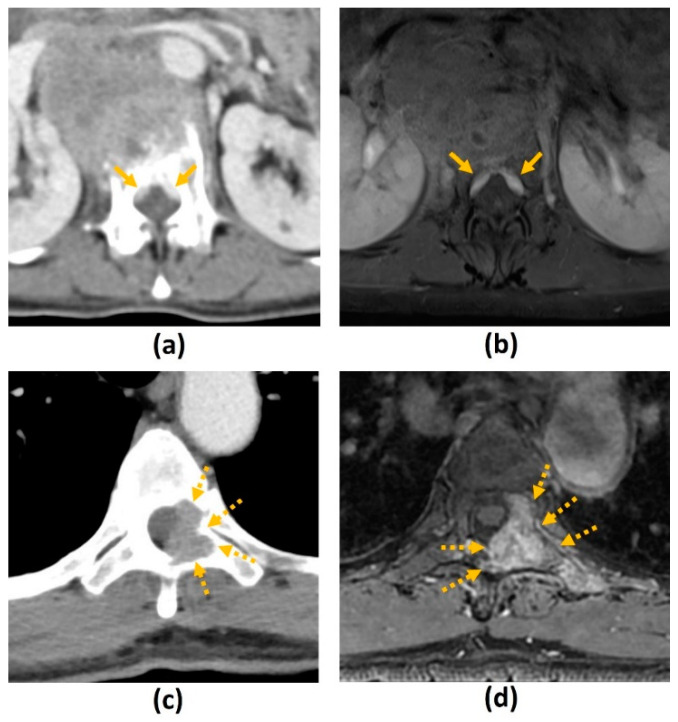
A 51-year-old rectal cancer patient with spine metastasis and concurrent inferior vena cava obstruction with resultant dilatation of the epidural venous plexus. Axial CT images in the soft tissue window (**a**), and axial post-contrast fat-suppressed T1-weighted (**b**) MR sequences of the spine. The dilated epidural veins (solid arrows) mimic an enhancing soft-tissue lesion in the epidural space and can be mistaken for metastatic spinal cord compression (MSCC). Another 68-year-old lung cancer patient with spine metastasis. Axial CT images in the soft tissue window (**c**), and axial post-contrast fat-suppressed T1-weighted (**d**) MR sequences of the spine. Enhancing epidural disease (dotted arrows) causes high-grade (Bilsky 2) MSCC.

MSCC is detected on CT as an amorphous enhancing soft tissue lesion in the epidural space which indents upon the thecal sac and/or spinal cord, depending on the Bilsky grade [23,70]. A potential pitfall is seen when there is dilatation of the epidural venous plexus, which can mimic enhancing epidural metastasis [71] (Figure 6). Other ways that MSCC may present on CT include paravertebral fat infiltration, vertebral collapse/deformity and rarely, malignant periosteal reaction of the involved vertebral body [70,72,73].

A retrospective study by Pezaro et al. (2015) on MSCC patients with metastatic prostate cancer showed that epidural disease was already discernible on CT performed a median of 28 days prior to the MRI study that diagnosed MSCC, in 80% of their patients [70]. Another review by Crocker et al. (2011) explored the diagnostic performance of routine whole-body CT in diagnosing MSCC [72]. This study showed that CT had a high sensitivity (88.9%, range: 80–100%) and specificity (92%, range: 88.6–97%) for identifying and excluding MSCC. A Glasgow oncology centre had also noted that accurate identification of the level of cord compression by MSCC could be readily achieved using CT, in a study involving 13 MSCC patients who had contemporaneous CT at the time of MSCC diagnosis by MRI [74].

While MRI remains the gold standard of imaging for MSCC diagnosis, 24-h on-site access may not be readily available in all institutions. In emergent situations where rapid imaging diagnosis is needed, the aforementioned findings suggest that CT can be used to assess for MSCC. In addition, whole-body CT is frequently performed for staging purposes after the initial diagnosis of cancer and for response assessment after systemic therapy for metastatic disease. In our experience, it is not uncommon for CT to be the first imaging modality to detect vertebral metastasis and/or MSCC. The patients in the latter instance are usually imaged prior to the onset of neurological deficits, and this may provide a valuable opportunity for early diagnosis and treatment.

Another role of CT imaging in MSCC patients lies in pre-SBRT planning as it can visualize bony anatomy well and assess the electron density of tissues which is important for dose distribution calculation [26,62,75]. It is also useful for determining the integrity of cortical bone for pre-operative planning for vertebroplasty, kyphoplasty and other spinal surgery in MSCC patients [1].

The limitations of CT lie mainly in its inferior soft tissue resolution compared to MRI. CT shows lower diagnostic accuracy than MRI in diagnosing MSCC [1] and vertebral body metastasis [23,24,26,76], and it does not have the ability to evaluate for spinal cord oedema/myelomalacia due to compression. Other limitations include radiation exposure, the presence of beam hardening artifacts and the potential confoundment of lytic metastasis by osteoporotic change [23].

#### 3.4.1. Challenges of CT: Metal-Related Artifacts

Metal-related artifacts in CT arise due to a combination of processes, primarily photon-starvation and beam hardening artifacts. Photon starvation artifacts occur due to metal having a comparatively higher atomic number than soft tissues. This results in significantly greater attenuation of photons by the photoelectric effect, reducing the number of photons reaching the CT detectors and reducing the SNR. Resultant streak artifacts around the implant occur, predominantly along the axis of greatest attenuation. Beam-hardening artifacts, on the other hand, occur due to the polychromatic nature of the X-ray beam in CTs, as lower energy photons are preferentially attenuated compared to higher energy photons. This alters the mean energy and energy distribution of the X-ray beam. It is more pronounced when the beam travels through materials with a higher atomic number including metals and calcium/bones. As CT utilizes X-ray beams of different projections, these different beams will consequently have different energy distributions as they travel through different thicknesses of material. This leads to inconsistent data acquisition, resulting in the appearance of dark streaks surrounding metal implants. Other contributors to metal-related artifacts include scatter artifacts, splay artifacts, and non-linear partial volume effects [37,77,78,79].

**Figure 7:**
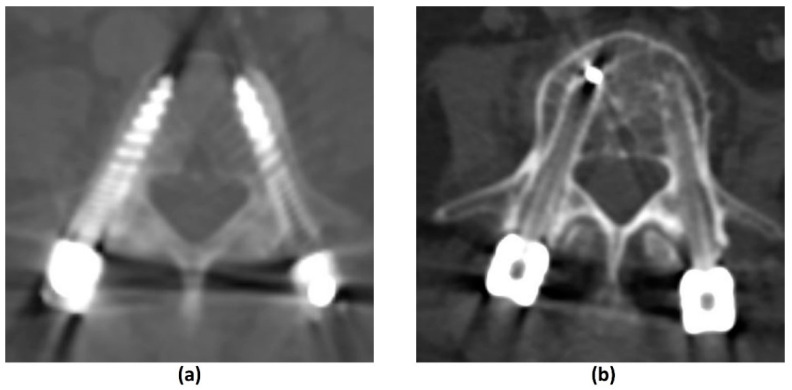
Titanium screw implants (**a**) and CFR-PEEK implants (**b**) on CT imaging. CFR-PEEK implants are radiolucent and result in less metal-related artifacts including beam hardening artifacts.

Similar to MRI, the extent of metal-related artifacts may also be affected by implant and hardware factors. Smaller metal implants with lower attenuation coefficients produce less artifacts. For example, a titanium (Z = 22) surgical clip will produce less artifacts than a platinum endovascular coil (Z = 78) [33,77]. CFR-PEEK implants have also shown benefits in MAR on CT (Figure 7). A comparison of standard titanium and CFR-PEEK spinal implants in sheep cadavers showed that of the two, CFR-PEEK implants produced less artifacts and demonstrated better artifact reduction than other scanning and image reconstruction MAR strategies including dual-energy CT (DECT) [78]. In addition, artifacts are most pronounced along the axis of maximal thickness of the implant. This can be overcome by repositioning the regions of clinical interest although this may be less feasible in spinal implants [33].

Hardware factors include scanning parameters, techniques, and various MAR algorithms. Increasing the X-ray peak voltage and tube current, as well as decreasing the pitch can reduce metal-related artifacts at the drawback of increasing patient dose. Decreased soft tissue contrast is also seen with increasing peak voltage. Other methods include the use of filtration and beam-hardening correction software, which are common in-built features to reduce beam hardening artifacts in modern CT scanners [33,37,77,79].

**Figure 8:**
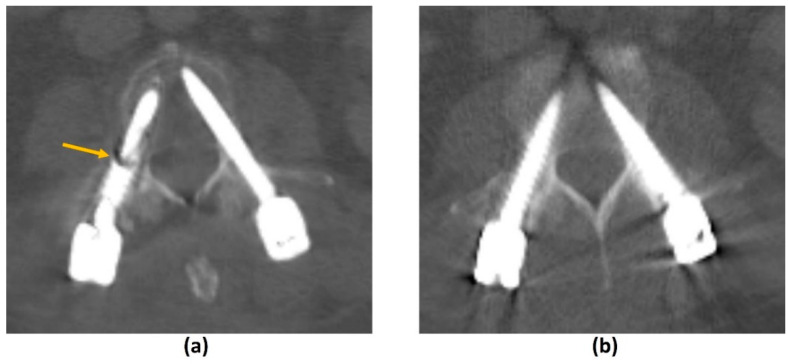
Axial CT-images in a 53-year-old patient with spinal implants with (**a**) and without (**b**) a post-processing metal artifact reduction (MAR) algorithm. While there is a noticeable reduction in metal-related artifacts with the MAR algorithm, there is an apparent linear lucency (solid arrow) across the right screw at the pedicle region. This was a new streak artifact inadvertently produced by the MAR algorithm and comparison with the non-MAR images is important to avoid mistaking these new artifacts as implant fractures.

Effective methods that do not increase radiation dose are post-processing projection-based MAR algorithms. Examples in commercial use are O-MAR (Philips Healthcare, Amsterdam, The Netherlands), iMAR (Siemens Healthineers, Erlangen, Germany), Smart MAR (GE Healthcare, Chicago, IL, USA) and SEMAR (Toshiba Medical Systems, Otawara, Japan) [80,81,82,83]. These algorithms work by using a Hounsfield unit (HU) cut-off (usually >3000 HU) to detect and segment metal in the uncorrected image. Forward-projection is performed to identify the corrupted projection data which is then removed. Estimations from uncorrupted projection data are then used to reconstruct the corrected image [37,77]. Projection-based MAR algorithms do not increase the radiation dose to the patient and can be applied retrospectively (in contrast to DECT). However, there is a potential loss of information due to the removal of metal-contaminated projection data. New artifacts can also be introduced into the final image with areas of pseudo-osteolysis at the bone-metal interface, residual or new bright and dark streak artifacts, and apparent disappearance of the metal implant or reduced implant size [37,77,79,84,85] (Figure 8).

While projection-based MAR algorithms address artifacts related to photon starvation, DECT can be utilized to reduce beam hardening artifacts without significant additional radiation exposure [37,77,86,87]. In DECT, two datasets of the same anatomic region are acquired with two different energy spectra that have different peak voltage settings. The data is then used to reconstruct a virtual monochromatic image—choosing a high peak voltage setting (around 95 and 150 keV) would reduce beam hardening artifacts at the expense of reduced soft tissue contrast and iodinated contrast enhancement [37,77,86,87]. Other voltage settings may be selected for different clinical purposes. For example, 140 keV facilitates assessment for prosthetic loosening while 70–80keV facilitates visualization of surrounding soft tissues and fluid [37]. Another benefit of DECT is that the virtual non-calcium algorithm can be used to enhance detection of bone metastasis in MSCC [88,89], even for the purposes of biopsy imaging of isodense lesions [90].

A study by Andersson et al. (2015) compared the performance of four commercially available CT MAR algorithms with DECT by imaging a phantom with bilateral hip prostheses. Findings showed that MAR algorithms had superior performance in metal-related artifact reduction than monochromatic reconstructions from DECT [79]. In another study, different implant types were shown to respond differently to DECT and projection-based algorithms with varying MAR efficacy; a combination of the two provided the best artifact suppression from spine implants [91]. While these MAR techniques are useful for follow-up imaging post instrumentation in MSCC, a recent systemic review showed that they have a limited role in radiotherapy applications due to various factors including the introduction of additional artifacts, and more research is needed to overcome these limitations [92].

#### 3.4.2. Photon-Counting CT

Photon counting CT is an emerging technology which utilizes energy-resolving detectors that detect the individual incoming photons and measure their energy. It eliminates the intermediate step of converting X-ray photons to light energy before the final conversion to an electrical output. It offers various benefits including improved spatial resolution, decreased noise and reduced radiation exposure. Importantly, it reduces beam hardening artifacts. MAR algorithms utilizing photon counting CTs have been proposed [93].

### 3.5. Other Imaging Modalities

#### 3.5.1. Plain Radiograph

Radiographs are inexpensive and readily available with low radiation exposure. However, they are generally insensitive for screening of asymptomatic bony metastasis and more than 50–70% of trabecular bone must be destroyed before an intramedullary tumour becomes apparent [24,94,95]. The two-dimensional nature also limits the amount of information that can be gleaned from a plain radiograph. The contents of the spinal canal are not well evaluated, although the likelihood of spinal cord compression can be inferred from secondary signs, including a pathological fracture with retropulsion. The 2008 NICE guidelines do not recommend using plain radiographs to confirm or exclude the diagnosis of spinal metastasis or MSCC [1].

#### 3.5.2. Skeletal Scintigraphy

The role of skeletal scintigraphy lies more in the detection of bone metastasis rather than in the detection of MSCC [1]. Tc-99m bone scans have the advantage of assessing the entire skeleton for detection of polyostotic disease, with sensitivity and specificity of 78% and 48% respectively. It has been reported to identify metastatic disease 2–18 months before they are apparent on radiographs. The disadvantages include a poor resolution, low specificity and poor sensitivity for predominantly osteolytic lesions [94,95].

#### 3.5.3. Positron Emission Tomography (PET)/CT and PET/MRI

Positron emission tomography (PET) fused with CT images generally improves the sensitivity of CT in detecting osseous metastasis and epidural disease, due to the earlier detection of increased glucose utilization in neoplastic cells in 18F-FDG PET/CT imaging compared to cortical destruction in CT [96]. 18F-FDG PET/CT shows comparable sensitivity to MRI in detecting bony metastasis, but this decreases with a smaller lesion size and decreased metabolism [97]. Note should also be made that osteoblastic tumours may have reduced glucose affinity, which may affect the sensitivity of detecting vertebral metastasis [98].

18F-FDG PET/CT may have a role in distinguishing benign from malignant vertebral compression fracture, with at least three different studies showing significantly increased standardised uptake value (SUV) in malignant vertebral compression fractures compared to benign compression fractures [99,100,101]. Proposed SUV threshold values have ranged from 3.45 to 4.25 [100,101]. The pattern of SUV uptake has also been shown to differentiate between the two, with a “striped” pattern seen exclusively in benign compression fractures in one of the studies [100]. Importantly, 18F-FDG PET/CT has a high sensitivity in detecting malignant compression fractures but inferior specificity, reportedly as low as 29% [101]. Reasons for false positives include bone marrow-stimulating agents and acute compression fractures, with uptake returning to normal in approximately 3 months after the inciting fracture [60,99]. It is suggested that 18F-FDG PET/CT may play a role in clinical decision-making when CT and MR findings are inconclusive [60]. Gwak et al. (2006) had also found it feasible to incorporate 18F-FDG PET/CT for radiosurgery planning in patients with recurrent spinal metastasis that was obscured by spinal implants on MRI and CT [102].

With regards to the diagnosis of MSCC, PET/CT provides limited evaluation of the contents of the spinal canal and generally does not provide any additional clinically relevant information over MRI [1]. The recent development of PET/MRI appears promising, however one should also be cognizant of the potential limitations of this imaging modality. This includes a relative lack of personnel (physicians and technicians) trained in both nuclear medicine and MRI interpretation, as well as the longer imaging time with MRI compared to CT. In addition, most PET/MRI studies currently utilize whole-body MRI images for localization. The large field-of-view limits the resolution and anatomical information provided, and patients may feel claustrophobic due to the whole-body surface coil [103,104]. It is hoped that with future research (e.g., acceleration techniques in MRI acquisition), some of these limitations may be overcome.

## 4. The Role of Imaging in Stereotactic Body Radiotherapy (SBRT)

Radiotherapy is one of the definitive local treatment options for MSCC and includes cEBRT and SBRT. SBRT is technically feasible in low-grade MSCC (up to Bilsky 1c) [105] and is mainly indicated in the setting of oligometastatic disease, re-irradiation, and in the treatment of radioresistant primary tumors where durable local control and symptom relief are required [106].

SBRT is a high-precision technique that relies on the accurate anatomical delineation of the tumour and adjacent critical structures to deliver highly conformal ablative doses while minimizing radiation to the surrounding organs at risk due to a steep dose gradient. This is achieved with highly precise and accurate image guidance in both SBRT planning and delivery [12,107,108,109,110].

The delivery of higher dose per fraction enables a shorter treatment time and allows the effective treatment of tumours previously considered radioresistant to cEBRT [12,17,107,111,112]. The effectiveness of SBRT in local tumour and symptom relief has been widely studied with favourable results [98,111,112,113,114,115,116]. A systemic review by Gerszten et al. showed that SBRT can achieve local control rates of approximately 90% and pain improvement rates of 85% [107], superior to that of cEBRT.

The role of imaging in SBRT lies in three main areas:

1. Pre-treatment planning;
2. In-room imaging guidance;
3. Post-treatment follow-up.

### 4.1. Pre-Treatment Planning

The Spine response assessment in Neuro-Oncology (SPINO) group recommends performing a high-resolution CT with slice thickness ≤ 2 mm for SBRT planning [98]. This allows depiction of bony structures with good spatial accuracy, metastatic lesion characterization and assessment of electron density of tissues. Due to the inferior soft tissue resolution in CT, there can be limited information on the tumour extent (particularly in the para-spinal and epidural areas). Moreover, accurate segmentation of the spinal cord is critical for SBRT, and limiting the cord dose is prioritized during SBRT planning. The SPINO group therefore recommends performing volumetric thin slice (≤3 mm) axial T1-weighted pre- and post-contrast and T2-weighted MRI close to the date of CT-simulation (ideally, within 1 week of simulation) [98].

**Figure 9:**
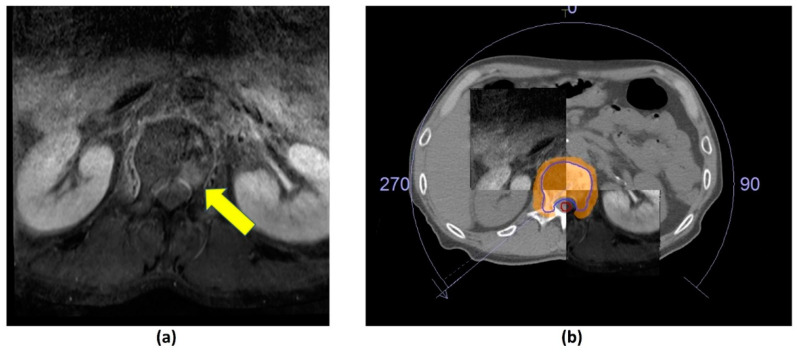
Axial post-contrast fat-suppressed T1-weighted MRI. (**a**) Metastatic lesion in the L1 vertebral body (yellow arrow). (**b**) CT and MR fusion for stereotactic body radiotherapy (SBRT) planning; 27 Gy over 3 fractions delivered using volumetric modulated arc therapy. Clinical target volume (CTV—blue outline), planning organ at risk volume (PRV, cord—red outline), 95% isodose (orange colour wash).

Isotropic volumetric MRI acquisitions enable multiplanar reconstructions to facilitate co-registration between the MRI and CT (Figure 9) [117]. Whenever possible, the treatment position should be replicated during the time of planning MRI, so as to facilitate accurate co-registration. Despite these measures, perfect co-registration is usually not achieved, due to spatial distortion artifacts commonly encountered in MRI [118,119].

Rogé et al. (2022) recently demonstrated that semi-automated clinical target volume (CTV) generation for SBRT planning of spinal metastasis had favourable accuracy when compared to manual contouring, and did not show a significant dosimetric increase to the organs at risk [120]. It is anticipated that future research would focus on the automation of tumour contouring, an otherwise laborious task if performed manually [121].

### 4.2. Image-Guided Radiotherapy (IGRT)

Image-guided radiation therapy (IGRT) for SBRT requires three-dimensional positional verification of the treatment isocentre prior to SBRT delivery. There are several points where anatomic or positional deviation may occur in radiation therapy, affecting the precision and accuracy of dose delivery. Daily set-up errors and deviations between the patient anatomy at pre-treatment planning and at the treatment itself (e.g., tumour growth, patient weight loss) are referred to as interfractional variation. Movement occurring in the course of a treatment session (e.g., patient motion and inherent cord motion secondary to the cardio-respiratory cycle) is referred to as intrafractional movement [117,122,123]. Inherent cord motion may account for up to 0.7 mm in the axial direction [124].

Failure to account for inter- and intrafractional motion can lead to adverse effects including underdosing the target volume, and/or harmful dose delivery to adjacent radiosensitive organs [122]. This is particularly important in SBRT treatment for less severe MSCC (e.g., Bilsky grade 1c), where the spinal cord lies in close proximity. Intrafractional movement may be increased in treatments lasting more than 20 min [125]. In such cases, our group recommends a mid-treatment CBCT to verify and correct for the treatment isocentre. A study by Oztek et al. (2020) had previously recommended a planning organ at risk volume (PRV) margin of at least 1.5–2 mm around the spinal cord to account for intrafractional cord motion [126].

IGRT for SBRT is predominantly performed through an onboard kilovoltage cone-beam CT. This allows for sub-millimeter spatial resolution, and modern radiotherapy treatment couches are able to correct for any deviations in the treatment isocentre, along the translational and rotational axes, i.e., 6D correction [127,128,129].

In CBCT, a cone-shaped X-ray beam is used with reciprocal two-dimensional area detectors instead of the collimated fan-shaped X-ray beam with the one-dimensional linear group of detectors that is used in conventional diagnostic CT. This enables volumetric acquisition with just a single rotation of the gantry without patient motion, compared to the helical acquisition of data seen in conventional CT imaging. The resultant three-dimensional volumetric data set can be reconstructed in all three orthogonal planes and is compared to the planning CT to calculate and correct for changes in target position prior to each radiation session [127,128]. It has to be noted that the time required for on-board CBCT acquisition (approximately 3–5 min) is considerably longer than conventional diagnostic CT. This limitation is predominantly due to the speed of gantry rotation.

Compared to conventional CT, CBCT has a reduced radiation dose [128,130]. However, there is increased scatter, beam hardening and other artifacts, causing a grainy and nonuniform appearance of the image [127,131,132,133,134]. These result in reduced soft tissue contrast and CT number accuracy [131,132,133]. Several strategies have been employed to overcome the artifacts in CBCT. For example, Bowtie filters and correction software have been employed to overcome beam hardening artifacts, motion artifacts have been reduced by patient immobilization and instruction to keep still, and other reconstruction and post-processing algorithms have been developed to tackle various other artifacts to improve image quality [127,133,134]. Preliminary research has also explored the role of dual-energy CBCT in overcoming these artifacts [132].

Other advanced imaging strategies in IGRT employ the use of onboard MR imaging, for example, Co60 MRI-guided radiotherapy and the more recent MRI-guided linear accelerator (MRI-LINAC) [135,136]. Unlike two-dimensional planar imaging and CBCT, MR-guided radiotherapy does not contribute to radiation dose. It also has the added benefit of being the imaging-of-choice for MSCC. Due to its superior soft tissue contrast, there are reduced safety margin requirements [137,138]. Other advantages include the ability of MRI to provide functional information on the tumour during the course of SBRT (e.g., apparent diffusion coefficient hypointensity as a marker of cellularity from high mitotic rate) [137]. Processes to do away with the mandatory CT simulation are being explored, by using MRI data to estimate the electron density for dose calculation [137,139].

Importantly, MRI has the potential to achieve real-time image guidance of the spinal cord in MSCC patients [135,136]. This allows detection of intrafractional motion. Treatment can then be halted almost instantly for patient repositioning, which had been previously shown to correlate with positional changes of the spinal cord in the vertebral canal [140]. Previous methods to control intrafractional variation have mostly relied on periodic mid-treatment imaging [125,141]. Another strategy to mitigate intrafractional motion employs the use of flattening filter free (FFF) SBRT to improve the dose rate and thereby reduce treatment time [142].

### 4.3. Post-Treatment Follow-Up

The SPINO group recommends performing an MRI of the spine 2–3 months after SBRT, with an interval MRI every 8–12 weeks thereafter to assess response to treatment. The studies should be reviewed by a radiologist and radiation oncologist. Earlier imaging may be warranted if the patient develops new neurological deficits or increasing pain [98].

**Figure 10:**
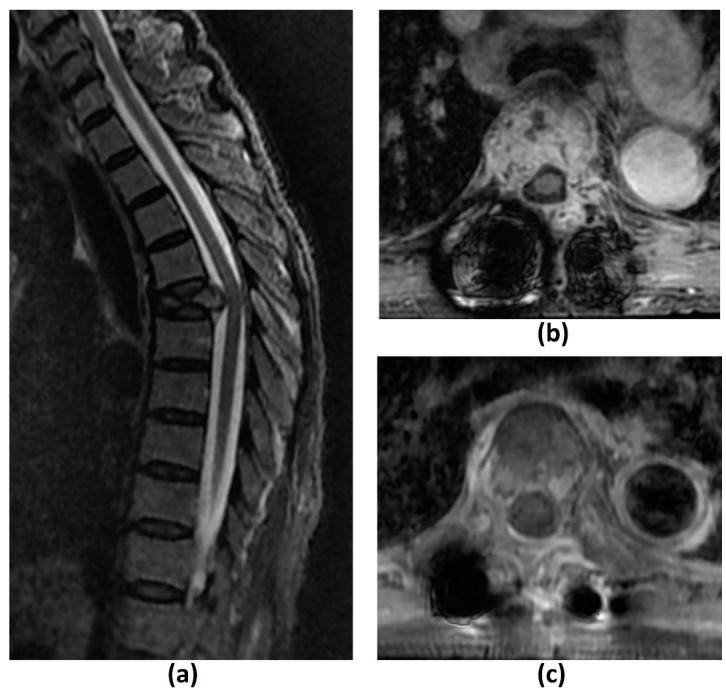
Sagittal T2-weighted image (**a**) of a 69-year-old female patient with metastatic rectal cancer to the spine shows a pathological T5 vertebral fracture. Axial post-contrast fat-suppressed T1-weighted images of the same patient, post separation surgery and planning for stereotactic body radiotherapy (SBRT) (**b**), and 6 months post-treatment (**c**). There is reduced tumour enhancement and bulk between (**b**) and (**c**) due to favourable post-SBRT response.

Local control is defined by a lack of disease progression within the treated region (Figure 10). This should be observed on two or three consecutive MRI studies that are performed 6–8 weeks apart. Local progression has several definitions. These include a definite expansion in tumour volume or increase in linear measurement, new epidural lesions and the presence of neurological deterioration with borderline increased extent of epidural disease on MRI [98].

Volumetric changes are a standard indicator of treatment efficacy of spinal lesions [117,143]. However, this may not be seen uniformly, even in patients who respond to SBRT. There is limited literature on the MRI appearances of bone metastasis post SBRT and it is still uncertain which signal changes are associated with treatment response or failure [98]. Hwang et al. (2011) studied changes in signal intensity of osteoblastic lesions post stereotactic radiosurgery, as these lesions normally show no volumetric changes during remission. Indicators of local control were shown to be increased T2-weighted signal intensities intermixed with areas of T2-weighted hypointensities [143]. This contrasts with another study by Zhou et al. (2014), which found that increased T2-weighted signal intensity was a predictor of local failure [144]. It should be noted that these studies had small sample sizes and further research is required.

The feasibility of functional imaging techniques (e.g., DCE MRI, diffusion-weighted MRI and PET/CT) in assessing treatment response of spinal metastasis post SBRT have also been studied. Kumar et al. (2017) showed that an increase in plasma volume on DCE MRI from pre- to post-radiotherapy was associated with local recurrence and a cut-off of −20% could predict local recurrence with high sensitivity and specificity. These changes were able to detect local recurrence up to 18 months earlier than conventional MRI [145]. The findings concur with a retrospective study by Chu et al. (2013), which also showed that reduction in plasma volume was the strongest predictor of treatment response [146]. Regarding DWI, Lee et al. (2021) found that the percentage change in ADC pre- and post-radiotherapy for bone metastasis from hepatocellular carcinoma was closely related to local tumour progression with a lower value predicting progressive disease [147]. Similarly, another study by Byun et al. (2002) showed that decreased DWI signal intensity of the bone marrow within metastatic spinal disease was indicative of successful radiation therapy [148]. Regarding PET/CT imaging, a preliminary study by Gwak et al. (2006) involving three patients with recurrent spinal metastasis treated with radiotherapy found that changes in SUV on 18F-FDG PET/CT correlated with clinical outcomes [102]. More recently, Choi et al. (2018) noted that >70% reduction in maximum SUV post SBRT in 42 patients with spinal metastasis who underwent 18F-FDG PET/CT before and after treatment was predictive of good progression-free-survival [149]. It should be noted however, that there is a possibility of a flare response post SBRT with variable increased radiotracer uptake that may persist up to 6 months [150]. While further research is needed to validate these findings, they suggest that post SBRT follow-up imaging may require a combination of functional and structural MRI for greater diagnostic accuracy.

Assessing for progression or regression in paraspinal and epidural disease is more straightforward. The former requires a definite change in volume and/or linear measurements, while the latter utilizes grading with the Bilsky criteria [21,98]. It should be noted that the most frequent site of disease progression post SBRT is the epidural space. This is due to planning criteria to limit the dose received by the spinal cord, and consequently underdosing the tumour abutting the spinal cord [98].

The role of CT in post SBRT follow-up imaging is complementary to MRI. It can assess the integrity of cortical bone, which can be affected by SBRT [98,117].

A common pitfall in post SBRT imaging is the phenomenon of pseudoprogression. This occurs when there is a significant increase in post-treatment lesion volume usually without significant clinical symptoms, with eventual stabilization or regression on subsequent imaging [117,151]. This is well documented in the brain, lung and liver [117]. These can be confused with true disease progression particularly if the patient experiences a pain flare, which reportedly affects 10–68% of patients post SBRT [152,153]. A biopsy may be warranted for a definitive diagnosis in such cases [98].

Pseudoprogression reportedly occurs in 14–18% of cases after spine SBRT [154,155]. A retrospective review by Bahig et al. (2016) found that pseudoprogression tended to be confined within the vertebral body while true local recurrences often involved the epidural space [155]. However, more research may be needed as a recent case report demonstrated a patient with pseudoprogression in the form of an epidural mass [156]. The timeframe is also important, with pseudoprogression occurring within a few weeks up to 6 months post SBRT, in contrast to the late presentation of radio-necrosis which may occur years after therapy [151]. Growth confined within the 80% prescription isodose-line and lytic lesions (as opposed to sclerotic lesions) have also been shown to be predictive of pseudoprogression [155,157].

VCF is a commonly encountered complication of SBRT [151]. The incidence rate of VCF ranges from 11–39%, with a systematic literature review by Faruqi et al. (2018) reporting a crude rate of 13.9% [158,159,160]. There is a median time of 2.6 months to VCF excluding outliers [158], as late-onset VCF of 2–3 years post SBRT have also been seen [161].

Both MRI and CT have limited ability to ascertain if a VCF was induced by SBRT or related to local tumour progression [162]. The SPINO group recommends histological confirmation in uncertain cases [98]. Al-Omair et al. (2013) had previously reported two patients who underwent spine SBRT, and subsequently developed imaging findings concerning for local tumour progression and VCF. However, biopsy eventually showed radiation-induced changes in the bone without evidence of tumour progression [161]. 

There are currently no established guidelines on the use of advanced imaging techniques/functional imaging like PET/CT or DCE MRI to distinguish between the two processes. While several studies have proposed a role of PET/CT in distinguishing benign and malignant compression fractures, these generally were not performed in the post-SBRT setting. Radiation-induced inflammatory changes may result in variably increased uptake on PET/CT in the immediate 6 months post SBRT and limit its diagnostic utility in such settings [150,162]. On perfusion/DCE MRI, vertebral metastases with or without associated pathological fracture were noted to demonstrate a significantly steeper enhancement slope and greater peak enhancement percentage than chronic compression fractures in a previous study involving 42 patients by Chen et al. (2002). However, no significant difference was found with acute compression fractures [163]. Further research into the role of functional imaging in evaluating VCF post SBRT is warranted, given the potential morbidity associated with salvage therapies. Future research may also focus on tools that predict the risk of VCF post SBRT treatment, so that prophylactic measures may be instituted. For example, Gui et al. (2021) had recently shown the feasibility of using the patient’s clinical information and radiomic features of the patient’s pretreatment imaging to develop a model that predicts the risk of VCF one year post SBRT treatment [164].

## 5. Deep Learning (DL) in MSCC Imaging

Recent advances in DL may improve MSCC imaging and diagnosis.

**Figure 11:**
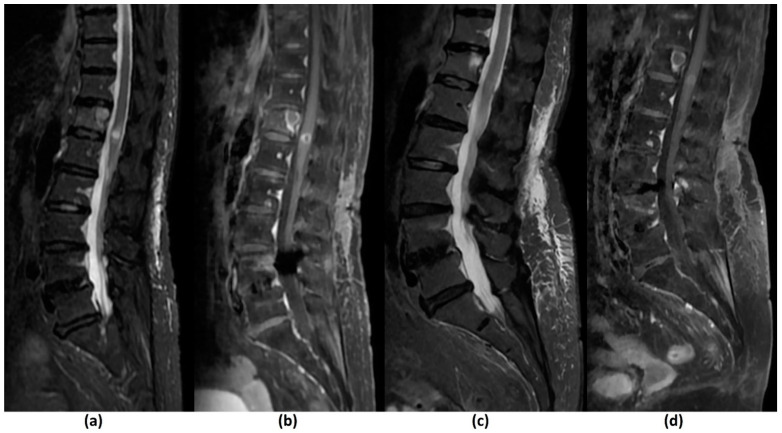

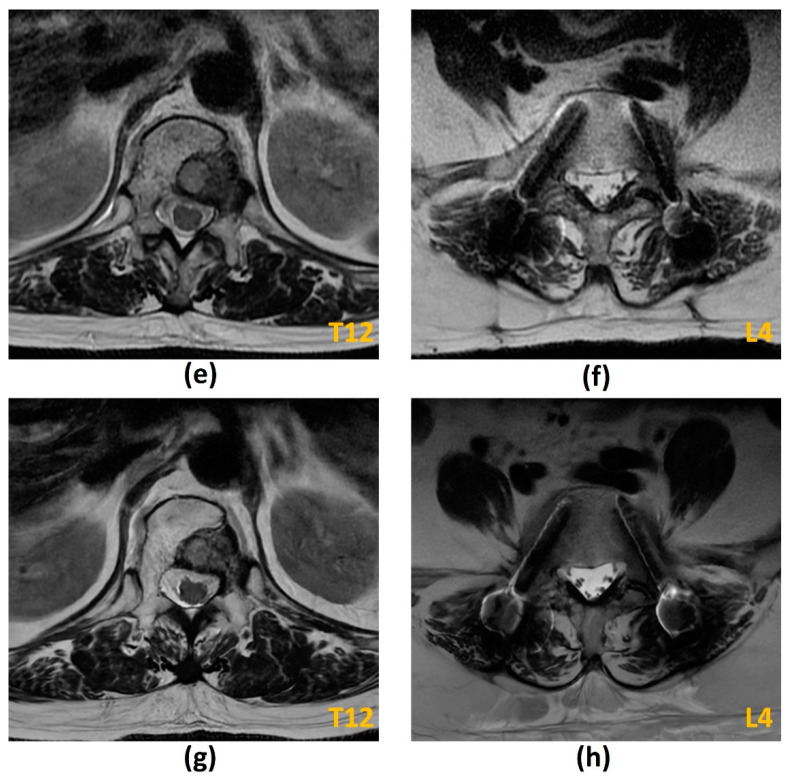
Spinal instrumentation with titanium screws in a patient with spinal metastasis. Standard sagittal STIR (**a**) and post-contrast fat-suppressed T1-weighted (**b**), and axial T2-weighted images at the level of T12 (**e**) and L4 (**f**). Six-month follow-up MRI was performed using AIR^TM^ Recon DL with sagittal STIR (**c**) and post-contrast fat-suppressed T1-weighted (**d**), and axial T2-weighted images at the level of T12 (**g**) and L4 (**h**). AIR Recon DL improves resolution and soft tissue contrast, significantly improving the diagnostic quality of images while reducing scan times.

AIR^TM^ Recon DL (GE Healthcare, Waukesha, WI, USA) [57,165] is a DL based MR reconstruction technique pioneered by GE Healthcare, which recently gained U.S. FDA 510(k) clearance (Figure 11). It utilizes trained neural networks to reduce scan times and improve image quality including SNR and spatial resolution. It also reduces ringing artifacts on MRI. These improvements in image quality will undoubtedly enhance the diagnosis of multiple pathologies on MRI, including MSCC. While a relatively new development, we anticipate that such techniques may have potential roles in MAR or IGRT in the future.

DL tools in image interpretation have seen substantial growth over the past few years. In a recently published study by Hallinan et al. (2022) [166], axial T2-weighted images of 177 MR spine studies in MSCC patients from Sept 2007 to Sept 2017 were utilized to create a DL model employing convolutional neural networks for automated MSCC classification into dichotomized Bilsky gradings (low-grade Bilsky was defined as Grade 0 to 1b, while high-grade Bilsky was defined as Grade 1c to 3). Internal testing on 38 MRI spine studies and external testing on 32 MRI spine studies showed near-perfect agreement of the DL model and other subspecialist readers (including a musculoskeletal radiologist, neuroradiologist, spine surgeon and radiation oncologist, all with at least 5 years of clinical experience) with the reference standard (internal testing kappas = 0.92–0.98, *p* < 0.001; external testing kappas = 0.94–0.95, *p* < 0.001).

These findings can potentially lead to significant time savings—automated detection of MSCC by the DL model can alert the radiologist and referring clinician, enabling prompt reporting and timely referrals. This is important given the rising demand for MRI studies and a shortage of radiologists worldwide [167].

The majority of the other DL models are centred on detection of vertebral body metastasis. For example, Wang et al. (2017) developed a DL tool for detection of spinal metastasis on MRI via deep Siamese neural networks. The model demonstrated a true positive rate of 90% and a false positive rate of up to 0.4 per case [168]. Another computer-aided detection system was developed by Hammon et al. (2013) for identification of osteoblastic and osteolytic metastasis on CT using a data set of 20 patients with lytic lesions and 30 patients with osteoblastic lesions [169]. Overall sensitivity was 83% and 88% for detection of osteoblastic metastasis and osteolytic metastasis respectively. False positives for osteoblastic metastasis were attributed to degenerative change and false positives for osteolytic metastasis were attributed to osteoporosis. O’Connor et al. (2007) developed a computer-aided detection system for detection of lytic thoracolumbar metastasis on body CT using a dataset of 50 patients, achieving a sensitivity of 94% on the test set, with a false positive rate of 4.5 per patient [170]. In addition, DL shows promise in quantifying skeletal metastatic burden on CT [171].

These studies show that DL tools for automated detection of spine metastases and MSCC are generally feasible and have the potential to achieve great time savings. By reducing the time to MSCC diagnosis, more efficient patient referrals to appropriate specialties can be made.

In our institution, a substantial number of MSCC patients are identified on routine whole-body CTs prior to development of neurological deficits. This offers a window for early intervention. Work is currently underway to develop a DL model to detect MSCC on routine whole-body CT.

## 6. Conclusions

MSCC is a debilitating complication in cancer patients with spinal metastasis, and its incidence is expected to rise due to improving cancer treatments and survival [6,166]. As it is a time-sensitive diagnosis, prompt radiological evaluation within 24 h is necessary to avoid permanent neurological dysfunction. MRI is the gold standard for imaging. In addition to establishing the diagnosis, MRI can grade MSCC (Bilsky criteria), determine the presence of spinal instability (partial SINS score) and assess the vertebral column for other sites of metastatic disease. CT Myelogram is an alternative imaging modality if MRI is contraindicated. Conventional CT plays a complementary role to MRI by characterizing the metastatic lesion and evaluating cortical anatomy. CT has been shown to be an effective triaging tool for determining the urgency of MRI in institutions where 24-h MRI access is not available. Both MRI and CT encounter difficulties in imaging around metal, and various metal-artifact reduction techniques are currently being studied. Acceleration techniques are also being developed to reduce long scan times in MRI.

Radiotherapy, including cEBRT and SBRT, constitutes one of the major treatment arms of MSCC. Imaging plays an important role in SBRT for pre-treatment planning, in-room image-guidance, and post-treatment follow-up. Recommendations from the SPINO group have been made on the definitions of local progression and local control post SBRT. However, there remain inherent difficulties in determining treatment response due to factors like pseudoprogression, and the uncertainty in signal changes indicating treatment response in bone metastasis. Functional MRI may help in the latter.

VCF is the most common complication post SBRT, and may be related to local tumour progression or the SBRT treatment itself. Current imaging modalities like MRI and CT have limited ability to distinguish between these two processes, and further research into the role of functional imaging in evaluating VCF post SBRT is warranted.

Recent advances in DL tools in image acquisition and analysis have the potential to revolutionize MSCC management by improving detection of spinal metastasis and reducing time to MSCC diagnosis, allowing earlier institution of definitive therapy.

## Figures and Tables

**Table 1:** The Spine Instability Neoplastic Score (SINS). A total score of 0–6 indicates stability, 7–12 indeterminate (potential instability), and 13–18 instability. A surgical review is recommended for patients with a total score of 7 to 18. Adapted from Fisher et al. [22].

Element of Spine Instability Neoplastic Score (SINS)	Score
**Location**	
Junctional (occiput–C2, C7–T2, T11–L1, L5–S1)	3
Mobile spine (C3–C6, L2–L4)	2
Semi-rigid (T3–T10)	1
Rigid (S2–S5)	0
**Pain relief with recumbency and/or pain with movement/loading of the spine**	
Yes	3
No (occasional pain but not mechanical)	1
Pain-free lesion	0
**Bone lesion (typically assessed with CT)**	
Lytic	2
Mixed (lytic/blastic)	1
Blastic	0
**Radiographic spinal alignment**	
Subluxation/translation present	4
De novo deformity (kyphosis/scoliosis)	2
Normal alignment	0
**Vertebral body collapse**	
>50% collapse	3
<50% collapse	2
No collapse with >50% body involved	1
None of the above	0
**Posterolateral involvement of the spinal elements** **(facet, pedicle or costovertebral joint fracture or replacement with tumour)**	
Bilateral	3
Unilateral	1
None of the above	0

**Table 2:** Typical parameters used in our institution for an MRI of the whole spine on a 1.5T platform for MSCC assessment. W, weighted. FS, fat-saturated. STIR, short tau inversion recovery. TR, repetition time, TE, echo time. All scans were conducted in the supine position with a torso coil.

	Sagittal			Axial	
Parameters	T2-W	T1-W Pre and Post-Contrast FS	STIR	Axial T1-W Post-Contrast FS	Axial T2-W
TR (msec)	3500	500	4000	500	3500
TE (msec)	90	10	60	10	90
Section thickness (mm)	3.5	3.5	3.5	5	5
Gap (mm)	1	1	1	2	2
Field of view (mm^2^)	400 × 400	400 × 400	400 × 400	160 × 160	160 × 160
Matrix	448 × 384	448 × 384	448 × 384	320 × 224	320 × 224

**Table 3:** Advantages and disadvantages of various MRI sequences in MSCC diagnosis. W, weighted. FS, fat-saturated. STIR, short tau inversion recovery.

Sequence	Advantages	Disadvantages
T2-W	Evaluation of spinal cord and nerve root compression (‘myelogram-like effect’); Detection of cord signal changes (e.g., myelomalacia or oedema)	Suboptimal for evaluation of marrow replacing lesions
T1-W	Identification of marrow replacing lesions including metastasis; Useful for comparison with post-contrast sequences to identify true contrast-enhancement	Suboptimal for evaluation of spinal cord and nerve root compression; Peritumoural oedema may also appear hypointense on T1-W sequences, which may limit the accuracy of measurement of the true tumour size
T1-W post-contrast FS	Detection of enhancing vertebral metastasis, sites of leptomeningeal and intramedullary disease; Delineation of tumour extent including identification of the epidural component, and presence of foraminal or paraspinal extension; Determination of biopsy site of highest yield (if biopsy required)	Suboptimal for evaluation of spinal cord and nerve root compression
STIR	Identification of marrow replacing lesions including metastasis; More accurate measurement of true tumour size from surrounding peritumoural oedema than T1-weighted sequences; Identification of macroscopic fat in lesions	Suboptimal for detection of sclerotic vertebral metastasis without oedema

**Table 5:** Characteristics that may distinguish benign osteoporotic compression fractures from malignant vertebral compression fractures [24,26,59,60].

Benign Osteoporotic Compression Fracture	Malignant Vertebral Compression Fracture
Posterior retropulsion of bony fragments or a concave posterior border of the vertebral body	Expansile convex posterior cortex
Normal marrow signal intensity (or a well-demarcated regular margin separating the spared marrow and abnormal marrow within the fractured vertebra)	Reduced signal intensity on T1-weighted imaging reflecting an underlying marrow replacing process, particularly if the posterior elements are involved
Remains isointense post-contrast imaging	Heterogeneously increased enhancement of the vertebral body
Usually without involvement of the posterior vertebral elements	Involvement of the posterior elements
Presence of multiple compression fractures (with the notable exception of multiple myeloma)	Presence of other spinal metastasis
Presence of a T1-weighted and T2-weighted hypointense band (thought to represent cancellous bone compaction, fluid or gas-filled clefts)	Abnormal epidural or paraspinal soft tissue or enhancement

## Supplementary Materials

The following supporting information can be downloaded at: https://www.mdpi.com/article/10.3390/cancers14133289/s1, Figure S1: Flow Diagram of Search Methods.
