# Supplementary figures and images for: State-of-the-Art Imaging Techniques in Metastatic Spinal Cord Compression
Source: Cancers (Basel). 2022 Jul 5;14(13):3289. doi: 10.3390/cancers14133289 (PMC9265325; doi:10.3390/cancers14133289)

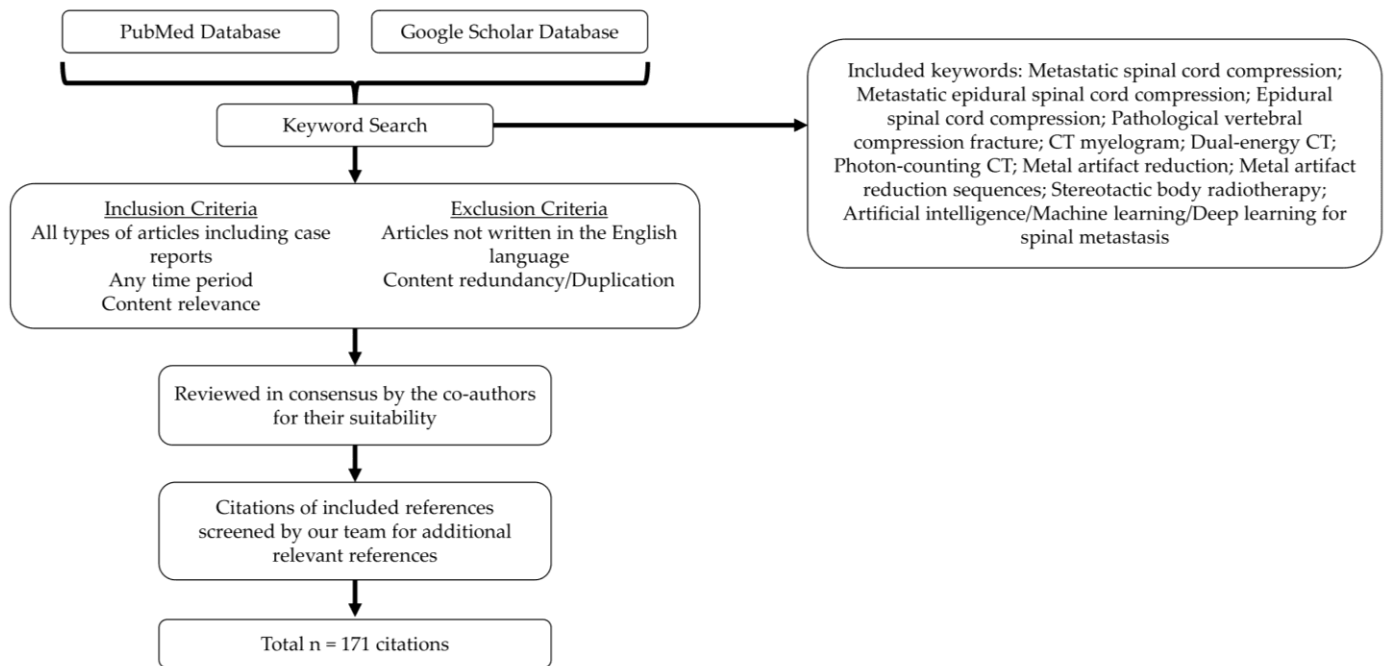

**Figure S1.** Flow Diagram of Search Methods.

Supplement: Supplementary file 1 [file cancers-14-03289-s001.zip › cancers-1776652-supplementary.pdf]
